# A six-long non-coding RNAs signature as a potential prognostic marker for survival prediction of ER-positive breast cancer patients

**DOI:** 10.18632/oncotarget.18919

**Published:** 2017-07-01

**Authors:** Lei Zhong, Ge Lou, Xinglu Zhou, Youyou Qin, Lin Liu, Wenqian Jiang

**Affiliations:** ^1^ Department of Breast Surgery, Second Affiliated Hospital of Harbin Medical University, Harbin 150086, China; ^2^ Department of Pathology, Second Affiliated Hospital of Harbin Medical University, Harbin 150086, China; ^3^ Department of PET/CT, Harbin Medical University Cancer Hospital, Harbin 150040, China

**Keywords:** breast cancer, estrogen receptor, long non-coding RNAs, prognosis

## Abstract

Dysregulated expression of lncRNAs has been observed in various human complex diseases (including cancers) by recent transcriptional profiling studies, highlighting potentials of lncRNAs as biomarkers for cancer diagnosis and prognosis. Despite some efforts have been made to search for novel lncRNA signature in breast cancer, the prognostic value of lncRNAs for ER-positive breast cancer patients still needs to be systematically investigated. In this study, we analyzed lncRNA expression profiles in a large of more than 600 breast cancer patients with ER-positive status from The Cancer Genome Atlas (TCGA) and identified six lncRNAs that are significantly associated with survival. Then a linear risk score model comprising six prognostic lncRNAs, termed six-lncRNA signature, was developed to identify high-risk patients from low-risk cases. The results of Kaplan-Meier analysis and ROC curves demonstrated the good sensitivity and specificity in survival prediction both in the training and testing datasets. Multivariate Cox regression analysis and stratified analysis showed that the six-lncRNA signature is an independent prognostic marker in survival prediction for ER-positive breast cancer patients. The GO enrichment analysis suggested that the six-lncRNA might involve with known breast cancer-related biological processes. With further experimental validation, these identified prognostic lncRNAs might have clinical implications for more personalized risk assessment for ER-positive breast cancer patients.

## INTRODUCTION

Breast cancer is one of the most frequent malignant cancers and the leading causes of cancer death in women [1]. Breast cancer patients can be classified into diverse patient subgroups with different prognosis and treatment response according to their classical clinicopathological and molecular features, such as estrogen receptor (ER), progesterone receptor (PR) and human epidermal growth factor receptor 2 (HER2) [2]. Estrogen receptor-positive (ER-positive) breast cancer is the most common type of breast cancer, accounting for almost 70% of cases diagnosed. Patients with ER-positive status are generally considered to have a better prognosis than those with ER-negative status [3]. However, approximately 30% of ER-positive patients, mainly due to the heterogeneous molecular characteristics of ER-positive patients, still faced a high risk of relapse within 10 years after surgery [4]. Therefore, the molecular signature was needed to identify ER-positive patients at high-risk for poor outcome who would benefit from systemic adjuvant therapy.

Recent advancements in RNA sequencing, cDNA cloning, and microarray technology have brought the discovery of thousands of long transcripts that were transcribed from thousands of loci in mammalian genomes and have no significant protein-coding capacity [5]. These long non-coding RNAs (lncRNAs) were defined as non-coding RNAs larger than 200 bp distinguishing from small ncRNAs, such as miRNAs [6]. Accumulating evidence suggests that lncRNAs played important roles both in the cell differentiation and developmental processes, such as dosage compensation, genomic imprinting, cell differentiation and organogenesis by controlling gene expression at transcriptional, post-transcriptional and epigenetic levels [7]. Dysregulated expression of lncRNAs has been observed in various human complex diseases (including cancers) by recent transcriptional profiling studies, highlighting potentials of lncRNAs as biomarkers for cancer diagnosis and prognosis [8–11]. Recent some studies have examined the roles of lncRNAs in cancer diagnosis and prognosis and identified several lncRNA-based molecular signature to predict patients’ outcome in some human cancers, including lung cancer [12–14], ovarian cancer [15–17], gastric cancer [18], glioma [19], oesophageal squamous cell carcinoma [20], diffuse large B-cell lymphomas [21, 22] and so on. Despite some efforts have been made to search for novel lncRNA signature in breast cancer [23–25], the prognostic value of lncRNAs for ER-positive breast cancer patients still needs to be systematically investigated.

In this study, we assess the prognostic value of lncRNAs by analyzing lncRNA expression profiles in a large of more than 600 patients with ER-positive status from The Cancer Genome Atlas (TCGA), and identified a novel six-lncRNA prognostic signature with the ability to predict the clinical outcome of ER-positive breast cancer patients.

## RESULTS

### Identification of lncRNAs associated with survival of patients with ER-positive breast cancer in the training dataset

We first performed univariate Cox regression analysis to examine the association between lncRNA expression and overall survival of patients with ER-positive breast cancer in the training dataset and identified 24 lncRNAs that are significantly associated with overall survival of ER-positive patients (*p* < 0.01). Then all these candidate prognostic lncRNAs were subjected to multivariate Cox regression analysis to consider their interactive effects, and a total of six lncRNAs were found to be independently correlated with patients’ overall survival (Table 1). Moreover, these six independent prognostic lncRNAs tended to be risky genes whose high expressions were associated with shorter survival.

**Table 1:** Detailed information of prognostic lncRNAs significantly associated with the overall survival in the training dataset

Ensembl version	Gene name	Position	Hazard^a^	Coefficient^a^	*p*-value^a^
ENSG00000224189	HAGLR	Chr 2: 176, 173, 195–176, 188, 958(−)	2.088	0.736	< 0.001
ENSG00000227477	STK4-AS1	Chr 20: 44, 963, 794–44, 966, 402(−)	1.389	0.329	0.002
ENSG00000237152	DLEU7-AS1	Chr 13: 50, 807, 856–50, 849, 905(+)	1.608	0.475	0.01
ENSG00000235314	LINC00957	Chr 7: 44, 039, 171–44, 042, 306(+)	1.711	0.537	0.025
ENSG00000230838	LINC01614	Chr 2: 215, 718, 043–215, 719, 424(+)	1.529	0.425	0.031
ENSG00000231249	ITPR1-AS1	Chr 3: 4, 490, 891–4, 493, 163(−)	1.499	0.405	0.039

^a^Derived from the univariate Cox proportional hazards regression analysis in the training dataset.

### Development of a six-lncRNA prognostic signature to predict overall survival from the training dataset

**Figure 1:**
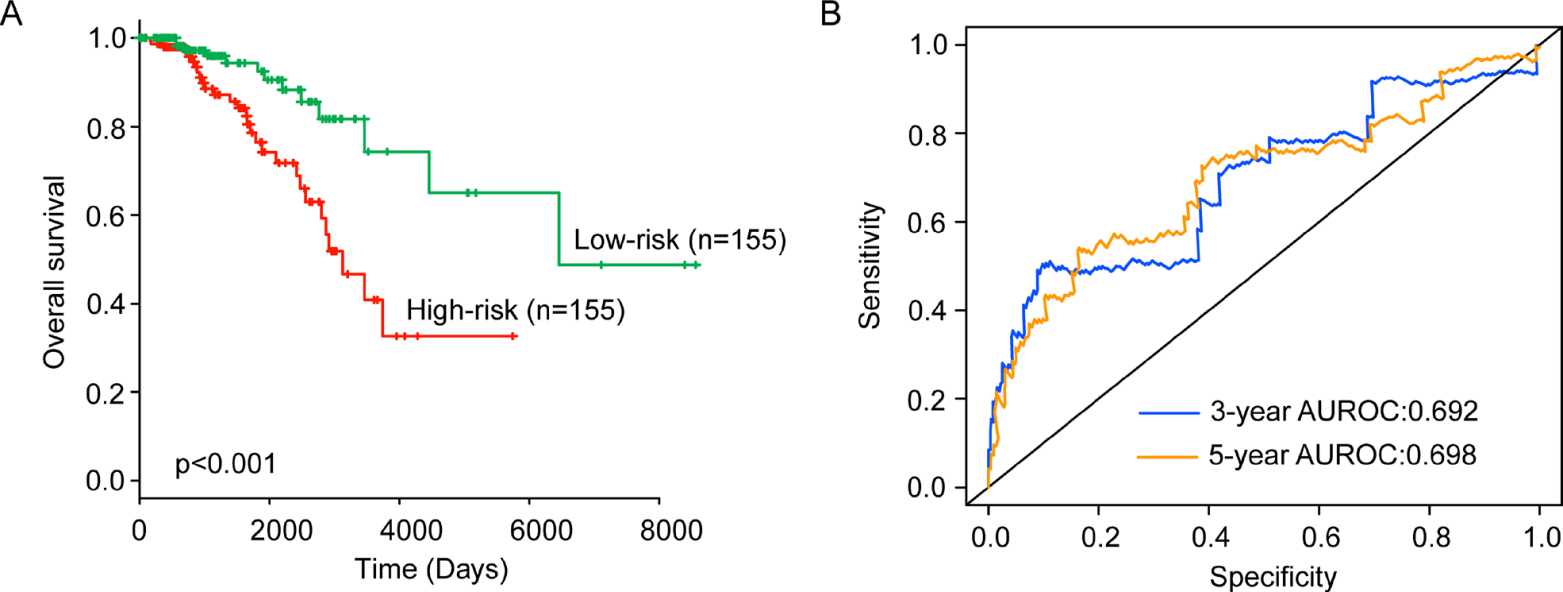
The performance of the six-lncRNA signature for survival prediction in the training dataset (**A**) Kaplan-Meier estimates of the overall survival between high-risk group and low-risk group in the training dataset. (**B**) ROC curves of the six-lncRNA signature at three and five years.

To build a lncRNA-based risk score predictive model, these six independent prognostic lncRNAs were subjected to multivariate Cox regression analysis to obtain their relative power in predicting overall survival. Then a six-lncRNA prognostic signature was developed by risk scoring method based on a linear combination of the expression levels of six independent prognostic lncRNAs, weighted by the coefficients derived from the multivariate Cox regression analysis as follows: Risk Score = ((0.5863* expression value of ENSG00000224189) + (0.2741* expression value of ENSG00000227477) +(0.3469* expression value of ENSG00000237152) + (0.6762*expression value of ENSG00000235314) + (0.4024* expression value of ENSG00000230838) + (0.2772* expression value of ENSG00000231249)). The six-lncRNA prognostic signature was first applied to 309 ER-positive patients of training dataset. A risk score was calculated for each of the patients in the training dataset based on the six-lncRNA prognostic model. Then all patients were classified into the high-risk group (*n* = 154) and low-risk group (*n* = 155) using the median risk score as risk cutoff value. Kaplan-Meier survival analysis and log-rank test suggested that there was a significant difference in overall survival between high-risk group and low-risk group (*p* < 0.001) (Figure 1A). Patients in the high-risk group tended to have significantly shorter overall survival time than those in the low-risk group (median survival 8.56 years vs. 17.69 years). The three- and five- survival rates of patients in the low-risk group are 95.9% and 92.5%, respectively, whereas corresponding rates in the high-risk groups is 88.6% and 76.4%. Moreover, the time-dependent ROC analysis for survival prediction of the six-lncRNA signature achieved an area under the curve (AUC) of 0.692 at three years and 0.698 at five years (Figure 1B). The results of the univariate Cox regression analysis showed that the expression levels of the six-lncRNA signature were significantly associated with overall survival of patients with ER-positive breast cancer in the training dataset (Hazard ratio (HR) = 1.543, 95% CI = 1.341–1.776, *p* < 0.001) (Table 2).

**Table 2:** Univariate and multivariate Cox regression analyses in the training, testing and entire TCGA datasets

Variables		Univariate analysis			Multivariate analysis		
		HR	95% CI of HR	*p*-value	HR	95% CI of HR	*p*-value
**Training dataset (*n* = 309)**							
Six-lncRNA signature	High/Low	1.543	1.341–1.776	< 0.001	1.455	1.257–1.685	< 0.001
Age	>= 59/< 59	1.029	1.006–1.053	0.015	1.019	0.995–1.044	0.117
Stage	(III/IV)/(I/II)	2.109	1.067–4.171	0.032	1.739	0.821–3.684	0.148
PR	+/−	1.604	0.628–4.1	0.323	1.156	0.443–3.014	0.767
**Testing dataset (*n* = 308)**							
Six-lncRNA signature	High/Low	1.127	1.071–1.458	0.036	1.189	1.166–1.525	0.017
Age	>= 59/< 59	1.053	1.027–1.08	< 0.001	1.062	1.033–1.092	< 0.001
Stage	(III/IV)/(I/II)	2.121	1.11–4.052	0.023	2.44	1.272–4.68	0.007
PR	+/−	0.356	0.165–0.766	0.008	0.421	0.182–0.971	0.042
**Entire TCGA dataset (*n* = 617)**							
Six-lncRNA signature	High/Low	1.474	1.305–1.666	< 0.001	1.473	1.284–1.690	< 0.001
Age	>= 59/< 59	1.041	1.024–1.059	< 0.001	1.046	1.028–1.065	< 0.001
Stage	(III/IV)/(I/II)	2.222	1.402–3.522	< 0.001	2.489	1.564–3.962	< 0.001
PR	+/−	0.836	1.305–1.666	0.541	0.724	0.391–1.340	0.303

### Validation of the six-lncRNA signature in the testing dataset and entire TCGA dataset

**Figure 2:**
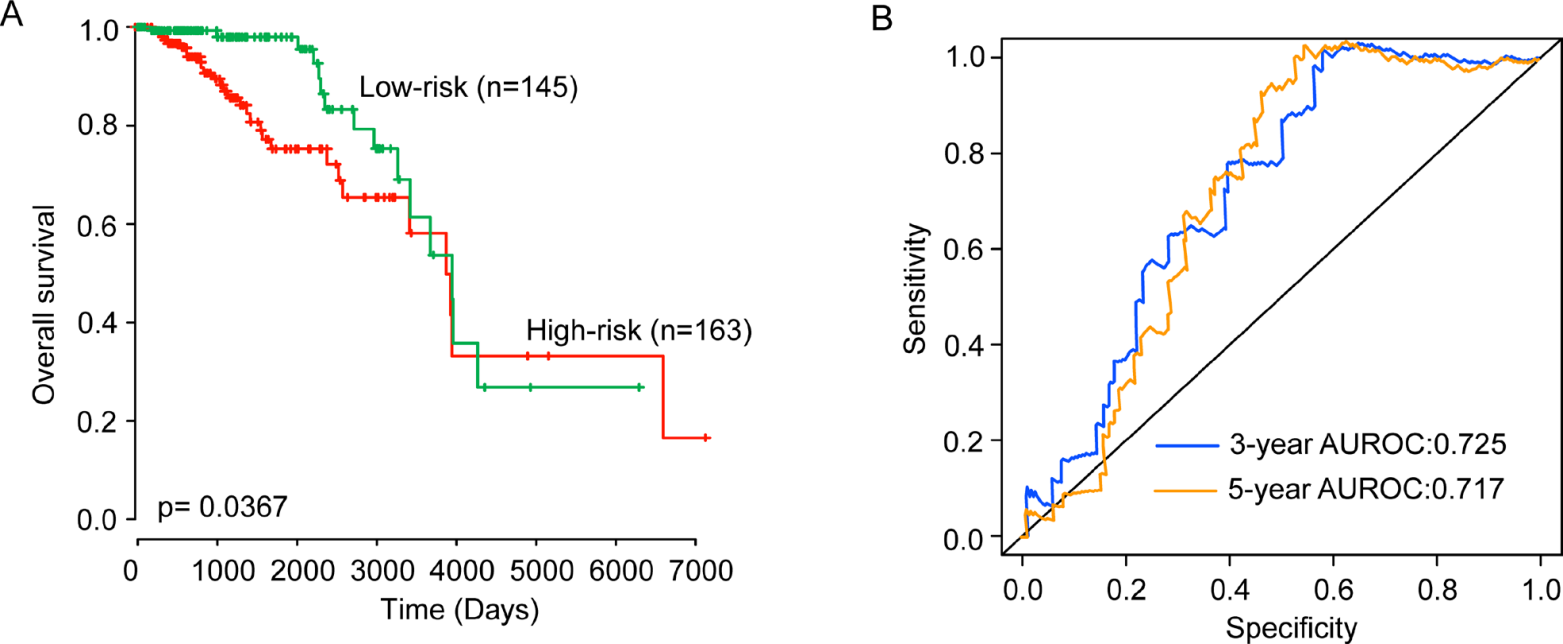
Validation of the six-lncRNA signature for survival prediction in the testing dataset (**A**) Kaplan-Meier estimates of the overall survival between high-risk group and low-risk group in the testing dataset. (**B**) ROC curves of the six-lncRNA signature at three and five years.

To test the prognostic value of the six-lncRNA signature in predicting overall survival of patients with ER-positive breast cancer, the six-lncRNA signature was tested in the testing dataset. By using the same risk score model, 308 patients of the testing dataset was classified into high-risk group (*n* = 163) and low-risk group (*n* = 145) using the same risk cutoff values for the training dataset. Consistent with the findings described above, patients in the high-risk group had significantly shorter overall survival than those in the low-risk group (median survival 10.61 years vs. 10.81 years, *p* = 0.037) (Figure 2A). The three- and five- survival rates of patients in the low-risk group are 98% and 98%, respectively, whereas corresponding rates in the high-risk groups are 86.9% and 75.3%. Moreover, the time-dependent ROC analysis for survival prediction of the six-lncRNA signature achieved an AUC of 0.725 at three years and 0.717 at five years (Figure 2B). In univariate analysis, the HR of high-risk scores versus low-risk scores for overall survival was 1.127 (95% CI = 1.071–1.458; *p* = 0.036) (Table 2), demonstrating a significant association between the six-lncRNA signature and patients’ overall survival.

**Figure 3:**
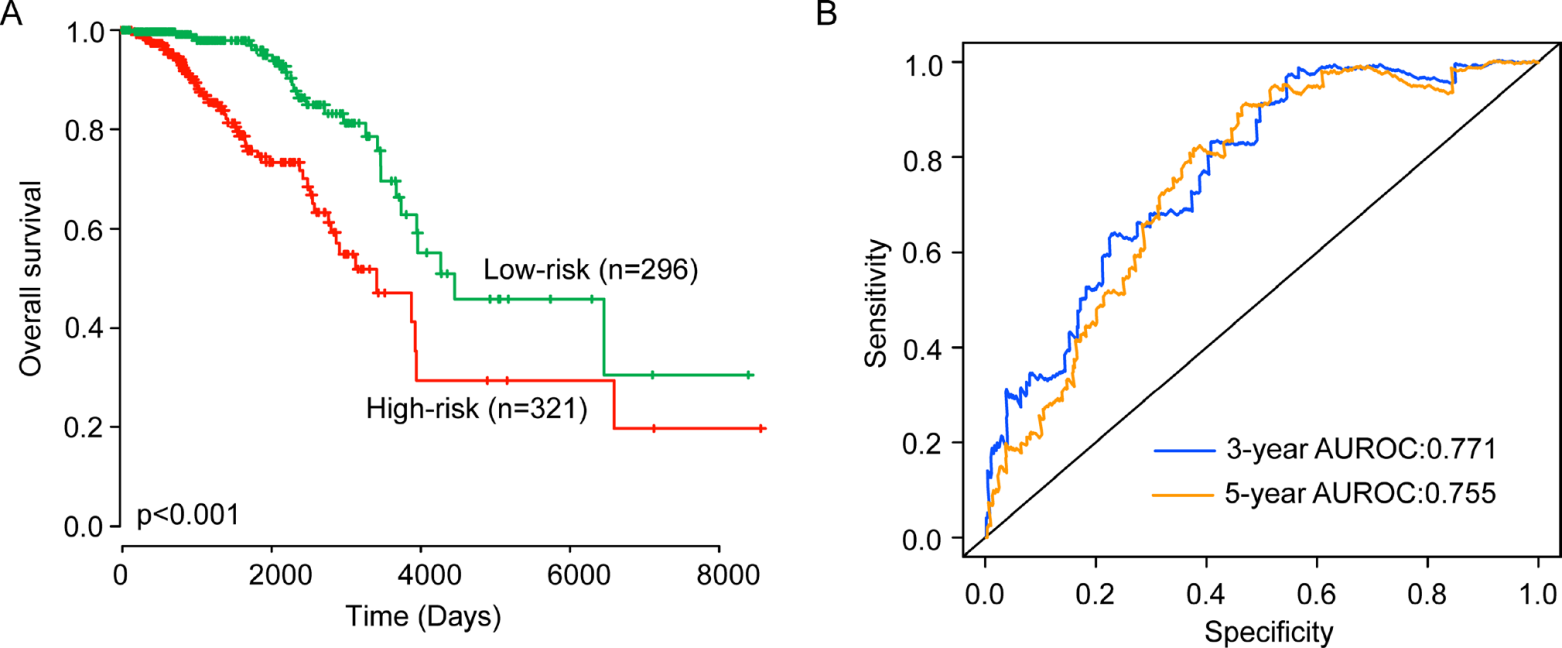
Validation of the six-lncRNA signature for survival prediction in the entire TCGA dataset (**A**) Kaplan-Meier estimates of the overall survival between high-risk group and low-risk group in the entire TCGA dataset. (**B**) ROC curves of the six-lncRNA signature at three and five years.

The six-lncRNA signature was further applied to all patients of the entire TCGA dataset to validate its predictive value. The same risk score model and risk cutoff criteria from the training dataset divided 617 patients of the entire TCGA dataset into the high-risk group (*n* = 321) and low-risk group (*n* = 296). The overall survival time of patients in the high-risk group was significantly shorter than that of patients in the low-risk group patients (median survival 9.34 years vs. 12.21 years, *p* < 0.001) (Figure 3A). The three- and five- survival rates of patients in the low-risk group are 97.9% and 96%, respectively, whereas corresponding rates in the high-risk groups are 86.8% and 74.5%. Validation of the six-lncRNA signature in the entire TCGA dataset of 617 patients produced a ROC with an AUC of 0.771 at three years and 0.755 at five years (Figure 3B). The HR of high-risk scores versus low-risk scores for overall survival was 1.474 (95% CI = 1.305–1.666; *p* < 0.001) in the univariate analysis (Table 2).

### Independence of prognostic value of the six-lncRNA signature from other clinical variables

**Figure 4:**
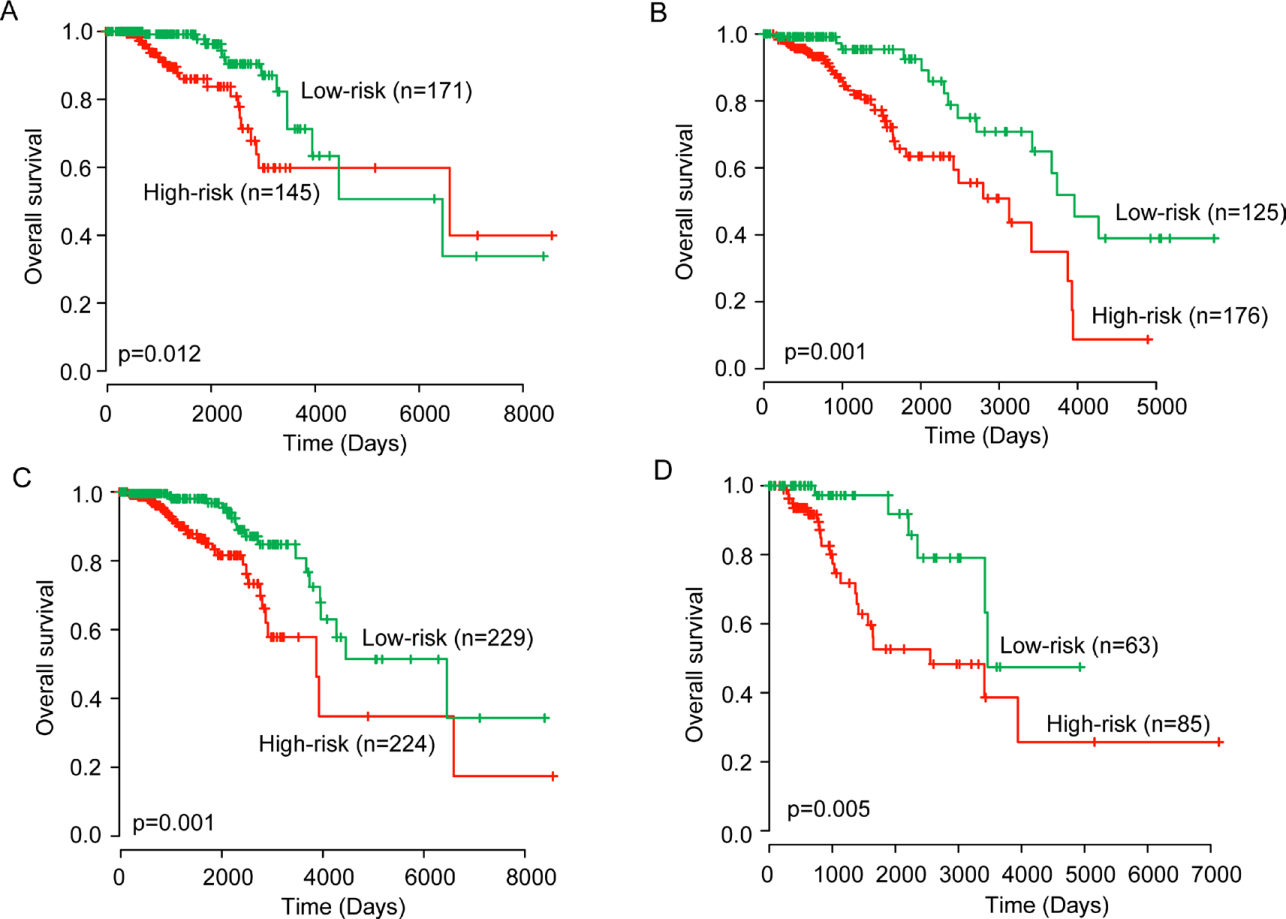
Kaplan–Meier estimates of the overall survival of patients with different clinical features Kaplan-Meier estimates of the overall survival between high-risk group and low-risk group for younger patients (**A**) and elder patients (**B**). Kaplan–Meier estimates of the overall survival between high-risk group and low-risk group for early-stage patients (**C**) and advanced-stage patients (**D**).

To assess whether the prognostic ability of the six-lncRNA signature is independent of other clinical variables of the patients with ER-positive breast cancer, multivariate Cox regression analysis was performed for each dataset including the six-lncRNA signature, age, stage and PR status. As shown in Table 2, the results of multivariate Cox regression analysis suggested that the six-lncRNA signature still maintained a significant correlation with overall survival after adjusted by age, stage and PR status (Table 2). The HR of high-risk group versus low-risk group for overall survival was 1.455 in the training dataset (95% CI = 1.257–1.685; *p* < 0.001), 1.189 in the testing dataset (95% CI = 1.166–1.525; *p* = 0.017) and 1.473 in the entire TCGA dataset (95% CI = 1.284–1.69; *p* < 0.001) when controlling for other clinical variables. However, we found that two clinical variables, age and stage, were significantly associated with overall survival in at least two of three patient datasets. So data stratification analysis was conducted according to age and stage. All patients were firstly stratified into a younger stratum (*n* = 297) and an elder stratum (*n* = 320). The patients in younger stratum or in elder stratum were classified into the high-risk group and low-risk group according to the six-lncRNA signature. As shown in Figure 4A and 4B, survival analysis suggested that patients in the high-risk group had significantly shorter overall survival than those in the low-risk group for younger stratum (*p* = 0.012) and elder stratum (*p* = 0.001), respectively. The same analyses were conducted in different stages showed that within each stage stratum, the six-lncRNA signature could further subdivide the patients into those likely to have longer survival and those likely to have shorter survival (Figure 4C and Figure 4D).

### Functional analysis of the six-lncRNA signature

**Figure 5:**
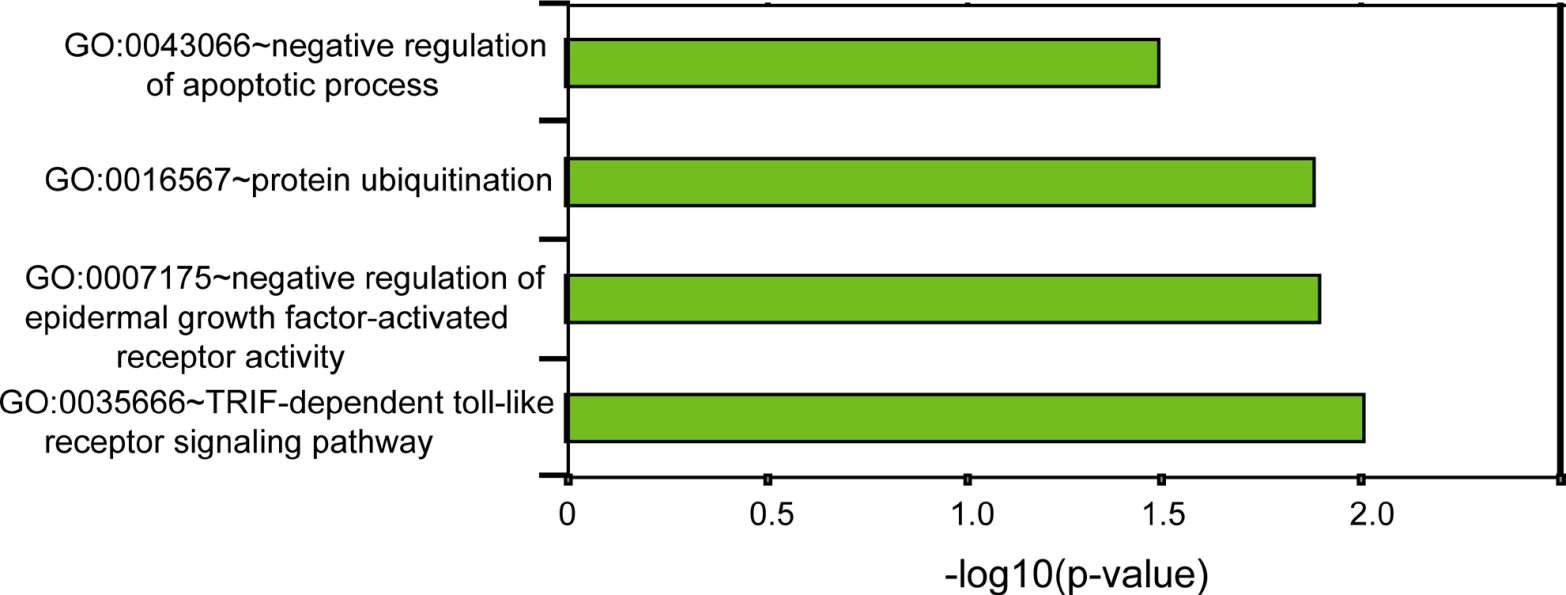
GO-based functional enrichment analysis

To gain a preliminary understanding of the six-lncRNA signature, we performed *in silico* analysis to infer potential functional roles of the six-lncRNA signature. We first examined the correlation between lncRNA expression and mRNA expression in patients of training dataset and identified 320 mRNAs that were positively or negatively correlated (top 1%) with that of at least one of the six prognostic lncRNAs as previously described [16, 26]. Then we performed GO function enrichment analysis for 320 co-expressed mRNAs and found that these 320 co-expressed mRNAs clustered most significantly in five GO biological progress, including TRIF-dependent Toll-like receptor signaling pathway, negative regulation of epidermal growth factor-activated receptor activity, protein ubiquitination, negative regulation of the apoptotic process (Figure 5).

## DISCUSSION

During the past decade, advances in clinical and molecular characteristics of breast cancer have demonstrated the heterogeneous features of breast cancer at the molecular and genetic levels [27]. Although improvement in the clinical management of breast cancer has led to a reduction in mortality rate, traditional clinical and pathological criteria is far from satisfactory largely due to molecular and genetic heterogeneity. It is well known that breast cancer could be divided into two subtypes according to estrogen receptor status: ER-positive breast cancers with a large number of estrogen receptors and ER-negative breast cancers without estrogen receptors. ER-positive breast cancer is the most common type of breast cancer and accounts for more than 70% of all breast cancers. Although patients with ER-positive breast cancer tended to have better survival than ER-negative patients, approximately 30% of ER-positive patients, mainly due to the heterogeneous molecular characteristics of ER-positive patients, still faced a high-risk of relapse within 10 years after surgery. Therefore, there is an urgently need to identify molecular markers for more personalized risk assessment for ER-positive breast cancer patients. Some efforts have been made to meet this need at mRNA and miRNA levels. Ahn and colleges assessed the prognostic values of 70-gene signature among patients with ER-positive breast cancer by analyzing expression data profiling from 186 patients with ER-positive breast cancer [28]. Zhou *et al*., revealed a 14-miRNA signature as a prognostic marker in ER-positive breast cancer by analyzing miRNA expression microarray data derived from TCGA project [29]. Another study performed by Philip *et al*. identified a novel MAPK-microRNA signature as novel predictive and prognostic biomarkers associated with poor clinical outcome [30].

Recently, a novel class of ncRNAs, termed lncRNAs, has been discovered in a large number of studies which have dramatically improved our understanding of cell biology and disease biology [31]. It has believed that lncRNAs is emerging as a novel player of cancer hallmark [32] and opens up a whole new range of possibilities for cancer diagnosis and prognosis prediction because lncRNA tended to be expressed in a more cell type- and tissue-specific manner and may be a direct indicator of tumor status compared to mRNAs and miRNAs [31, 33]. Dysregulated lncRNA expression has been observed in breast cancer tissue compared to normal breast tissue, highlighting the important roles in breast cancer carcinogenesis [34, 35]. Several lncRNA signatures have been identified as a prognostic marker for breast cancer. Meng et al. identified a four-lncRNA signature to predict breast cancer survival by analyzing four independent GEO datasets [25]. Another two lncRNA signatures also were recognized to be associated with survival of breast cancer patients using TCGA datasets [36, 37]. Zhou and colleagues revealed two lncRNA-related signatures to predict recurrence and metastasis of breast cancer patients [24, 38]. As mentioned above, breast cancer represents a highly heterogeneous disease and is made up of many subtypes. Moreover, comprehensive transcriptome analysis has found that there is specific lncRNA expression pattern within different subtypes of breast cancer. Therefore, there is an urgently need to identify lncRNA signature for more personalized risk assessment for ER-positive breast cancer patients.

In this study, we performed an integrated analysis of lncRNA expression and clinical features of 617 patients with ER-positive breast cancer from TCGA project which is supervised by the National Cancer Institute and the National Human Genome Research Institute. By using univariate Cox regression analysis followed by multivariate Cox regression analysis, we identified six prognostic lncRNAs significantly associated with clinical outcome of ER-positive breast cancer patients in the training dataset. A linear combination of these six prognostic lncRNAs was constructed as a novel lncRNA-based molecular signature to predict survival for patients with ER-positive status. This six-lncRNA signature was validated in the training dataset, testing dataset and entire TCGA dataset and demonstrated significant prognostic performance in three patient datasets. Among six prognostic lncRNAs, *HAGLR* is transcribed from the HOXD cluster on human chromosome 2q31.2 in an antisense manner [39]. Several groups have found that *HAGLR* is up-regulated in several human cancers (including breast cancer, bladder cancer, lung cancer and neuroblastoma) and the increased expression of *HAGLR* can promote oncogenesis via inhibition of apoptosis and is associated with the progression and unfavorable prognosis of these cancers [39–43]. These observations are consistent with our finding that the higher expression of *HAGLR* was associated with shorter survival of patients with ER-positive status. Another prognostic lncRNA, STK4-AS1, was recently proven to be regulated by STOX2-IT3-lncRNA associated with trophoblast differentiation and invasion [44]. Hu *et al*. also found that the prognostic lncRNA DLEU7-AS1 is overexpressed in mantle cell lymphoma (MCL) patient samples compared to normal B cells which can regulate SOX11 expression via PRC2 complex contributing towards the growth of MCL cells and as potential biomarkers for mantle cell lymphoma [45]. For the remaining three prognostic RNAs, to our knowledge, there is no available functional annotation. Our *in silico* GO enrichment analysis for co-expressed mRNAs suggested that variation in lncRNAs expression might affect critical biological processes involved in Toll-like receptor signaling pathway, epidermal growth factor-activated receptor activity, protein ubiquitination and apoptotic process which have been implicated in breast cancer tumorigenesis and development.

In conclusion, the present study analyzed the associations between lncRNA expression and survival of ER-positive breast cancer patients and identified a novel lncRNA signature comprising six lncRNAs (*HAGLR*, *STK4-AS1*, *DLEU7-AS1*, *LINC00957*, *LINC01614* and *ITPR1-AS1*) which can robustly predict the survival of breast cancer patients with ER-positive status. Moreover, the identified six-lncRNA signature demonstrated good performance in predicting three- and five-year survival and may be an independent prognostic marker in survival prediction for ER-positive breast cancer patients. The GO enrichment analysis suggested that the six-lncRNA might involve with known breast cancer-related biological processes. With further experimental validation, these identified prognostic lncRNAs might serve as alternative biomarkers and therapeutic targets for ER-positive breast cancer patients.

## MATERIALS AND METHODS

### Patient population and clinical information

Clinical information of patients with ER-positive breast cancer was retrieved from The Cancer Genome Atlas (TCGA) data portal (https://cancergenome.nih.gov/). After removing patients without clinical information and lncRNA expression profiles, a total of 617 patients with ER-positive breast cancer were used for further analysis. These 617 patients with ER-positive breast cancer were randomly divided into the training dataset (*n* = 309) and testing dataset (*n* = 308) for the purpose of discovery-validation. Detailed clinical information of patients with ER-positive breast cancer enrolled in this study was shown in Table 3.

**Table 3:** Summary of clinical characteristics of patients with ER-positive breast cancer in each dataset

Variables		Training dataset (*n*= 309)	Testing dataset (*n*= 308)	Entire TCGA dataset (*n* = 617)
Age, years, *n*(%)	>= 59	150 (48.5)	170 (55.2)	320 (51.9)
	< 59	159 (51.5)	138 (44.8)	297 (48.1)
PR status, *n* (%)	Positive	259 (83.8)	265 (86.0)	524 (84.9)
	Negative	48 (15.5)	42 (13.6)	90 (14.6)
	Unknown	2 (0.6)	1 (0.3)	3 (0.5)
Stage, *n*(%)	I/II	226 (73.1)	227 (73.7)	453 (73.4)
	III/IV	76 (24.6)	72 (23.4)	148 (24.0)
	Unknown	7 (2.3)	9 (2.9)	16 (2.6)
Survival status, *n*(%)	Alive	267 (86.4)	264 (85.7)	531 (86.1)
	Dead	42 (13.6)	44 (14.3)	86 (13.9)

### LncRNA expression profiles of patients with ER-positive breast cancer

LncRNA expression profiles were obtained from the TANRIC database (http://bioinformatics.mdanderson.org/) [46]. Briefly, RNA-seq BAM files of tumor patients were obtained from the UCSC Cancer Genomics Hub (CGHub, https://cghub.ucsc.edu/) and were used to quantify the expression levels of lncRNAs as reads per kilobase per million mapped reads (RPKM) [46].

### Statistical analysis

Univariate and multivariate Cox regression analysis were used to evaluate the association between lncRNA expression and survival and identify independent lncRNA biomarkers that significantly associated with survival. A lncRNA-based risk scoring predictive model was constructed by a linear combination of the expression values of independent lncRNA biomarkers and the multivariate Cox regression coefficient as the weight. With the lncRNA-based risk scoring predictive model, the patients with ER-positive breast cancer were classified into high-risk group and low-risk group using the median risk score of training dataset as the cutoff point. Survival differences between the low-risk and high-risk groups in the training dataset and testing dataset were assessed by the Kaplan-Meier survival plots, and compared using the log-rank test. The prognostic performance was measured using time-dependent receiver operating characteristic (ROC) curves. Univariate and multivariate analyses with Cox proportional hazards regression for survival were performed on the individual clinical variables with and without the lncRNA signature. Hazard ratios (HR) and 95% confidence intervals (CI) were calculated. All analyses were performed with R software.

### Function enrichment analysis

Functional enrichment analysis was performed using the DAVID Bioinformatics Tool (https://david.ncifcrf.gov/, version 6.8) which is widely used functional annotation tool for a gene set of interest. The results of enrichment analysis were obtained limited to GO terms in the “Biological Process” (GOTERM-BP-FAT) using the functional annotation clustering and functional annotation chart options with the human whole genome as background. The enriched GO terms with *p*-value < 0.05 were considered as a potential function of prognostic lncRNAs as previously described [13, 15, 47].
